# An Improved Sign Language Translation Model with Explainable Adaptations for Processing Long Sign Sentences

**DOI:** 10.1155/2020/8816125

**Published:** 2020-10-23

**Authors:** Jiangbin Zheng, Zheng Zhao, Min Chen, Jing Chen, Chong Wu, Yidong Chen, Xiaodong Shi, Yiqi Tong

**Affiliations:** ^1^Department of Artificial Intelligence, School of Informatics, Xiamen University, Xiamen 361005, China; ^2^China Mobile (Suzhou) Software Technology Co., LTD, Suzhou 215000, China; ^3^Department of Electrical Engineering, City University of Hong Kong, Kowloon, Hong Kong

## Abstract

*Sign language translation* (SLT) is an important application to bridge the communication gap between deaf and hearing people. In recent years, the research on the SLT based on neural translation frameworks has attracted wide attention. Despite the progress, current SLT research is still in the initial stage. In fact, current systems perform poorly in processing long sign sentences, which often involve long-distance dependencies and require large resource consumption. To tackle this problem, we propose two explainable adaptations to the traditional neural SLT models using optimized tokenization-related modules. First, we introduce a *frame stream density compression* (FSDC) algorithm for detecting and reducing the redundant similar frames, which effectively shortens the long sign sentences without losing information. Then, we replace the traditional encoder in a *neural machine translation* (NMT) module with an improved architecture, which incorporates a *temporal convolution* (T-Conv) unit and a *dynamic hierarchical bidirectional GRU* (DH-BiGRU) unit sequentially. The improved component takes the temporal tokenization information into consideration to extract deeper information with reasonable resource consumption. Our experiments on the *RWTH-PHOENIX-Weather 2014T* dataset show that the proposed model outperforms the state-of-the-art baseline up to about 1.5+ BLEU-4 score gains.

## 1. Introduction

Sign languages are visual-based natural languages used by the deaf people for their communication. Since most hearing people cannot understand sign language, *sign language translation* (SLT) has become an important application to bridge the communication gap between deaf and hearing people. In recent years, researchers have successively proposed deep learning models for neural SLT (e.g., [1–6]).

The existing SLT models basically follow a multimodal architecture, where *convolutional neural network* (CNN) and *neural machine translation* (NMT) are sequentially connected. The CNN module is used to extract image-level features, reduce the fine-grained input, and generate a tokenization layer as the input to the NMT module; the NMT module is the main translation module for encoding and decoding to generate target sentences. The above basic SLT architecture was first proposed by Camgoz et al. [1]. The tokenization layer serves as a hub layer in this architecture. Hence, optimizing it can improve the performance of both CNN and NMT.

However, most of the current SLT works only improve the CNN or NMT module separately, resulting in poor connection between the two modules which causes two serious problems:

1. Poor interpretability: most of the improvements focus on some common tricks, rather than considering the uniqueness of SLT. The characteristics of SLT determine that it is a special NMT task, although the input form is different from conventional spoken language. Therefore, analyzing from the input form may help us to find some interesting SLT phenomena and get a better interpretability. For a spoken sentence, the input is usually a series of words. Although there are semantic connections between words, they are expressed in a discrete form. As for a sign sentence, the input is usually a video signal. In actual application, the video needs to be framed into continuous frame images. Intuitively, we can compare each video frame to the basic word element of sign language. Unlike spoken language, the video frames of any sign sentence are continuous, and the order is closely related. In other words, it is illegal to reverse the order between any frames. Specifically, we found that there are many similar frames in the neighborhood, and these frames repeatedly express some meanings, which will cause redundant information and long sentence. However, no works use this visual phenomenon to custom optimization algorithms for sign language.
2. Poor performance for long sentences: longer sentences result in long-distance dependencies, large resource consumption, and low evaluation scores. This shows that both CNN and NMT modules need to be improved. However, the visual CNN module is obtained more attention, and the work of the innovative NMT module is obtained less attention. Besides, the improvement from the perspective of model interpretation is also a very important aspect.

Longer sentences mean more frames. The longer the sentence is, the more complicated the relationship between video frames will have, which leads to insufficient connection between frames. In theory, the amount of calculation may increase exponentially. Hence, the SLT model generally specifies a maximum number of input frames for the CNN module. For longer sentences, how to express more effective information within a certain window size is a meaningful research point. However, there is no work considering reducing useless frames from understandable visual features. Especially for longer sentences, CNN is more pressured and less efficient. If we can reduce the number of sign language frames according to the visual surface image features, then we may still get the same sentence meaning with a fewer frames (like turning long sentences into short sentences), which can not only reduce the convolution pressure, but also generate a higher quality tokenization layer. Moreover, the tokenization layer is then input into the NMT module, so optimizing it in the tokenization level will be a key role for improving the subsequent NMT.

**Figure 1:**
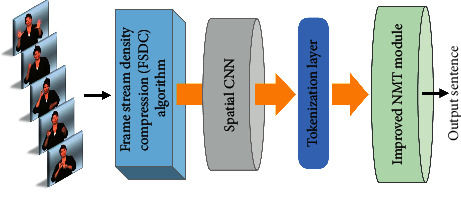
Overview of our proposed end-to-end SLT model with improved tokenization-related units, which includes an *FSDC* optimization algorithm and an improved NMT module.

To solve the above mentioned issues, we propose a novel SLT model with a better interpretability for longer sentences, as shown in Figure 1. There are two improvements with tokenization-related units.

First, we propose a frame-level *frame stream density compression* (FSDC) algorithm, which can compare pixels at the image level in an unsupervised manner, reducing redundant frames in temporal neighborhood. Intuitively, it can be understood as retaining high-density information by comparing the similarity of input image frames in the neighborhood. The reduced convolution information can generate tokenization with a smaller size, which allows more information to be transmitted within the limited window length. Besides, for the NMT module, reducing the number of input frames means a shorter length of input. Overall, this is a visually interpretable optimization of sign language that converts long sentences into short sentences.

Second, we replace the traditional encoder in the NMT module with an improved architecture to further strengthen the association between long sentence video frames. Inspired by the study of FairSeq [7], a hybrid model is proposed. The model incorporates a *temporal convolution* (T-Conv) unit and a *dynamic hierarchical bidirectional GRU* (DH-BiGRU) unit sequentially. It first convolves the input in the time domain and then encodes the semantic information in the subsequent deep hierarchical RNNs. We can still treat the tokenization layer as a vector representation layer of the dimensionality-reduced frames. As an improvement, 3DCNN/C3D was used in the CNN module [8, 9] to strengthen the association between frames in the time domain. However, it requires larger resource consumption and does not always work well in the case of low sign language resources. We observed that, if the NMT module convolves the sign sentences at the tokenized level in the time domain using 2DCNN, it can not only approach the function of 3DCNN/C3D, but also approach the speed of 2DCNN. All in all, this also shortens long sentences in the time domain and deepens the RNN structure in a hierarchical way. In this case, the NMT structure can handle longer sentences as easily as short sentences.

The main contributions of this paper are as follows:

1. We have proposed a novel SLT model with tokenization-related units, which can better handle longer sentences in lower resource consumption, and has a better interpretability.
2. We have introduced for the first time an unsupervised FSDC algorithm to compress the density of the input frames without removing key information. This method is suitable for many similar video tasks.
3. We have proposed a novel NMT module for SLT with optimized encoder-related units, *temporal convolution and dynamic hierarchical bidirectional GRU hybrid network* (TC-DHBG-Net), which compresses the effective information of the tokenization layer from the time domain so that long sentences are further shortened on the time domain to facilitate hierarchical GRUs to find semantic information.
4. Moreover, our improved neural SLT model has been made publicly available (https://github.com/binbinjiang/nslt_xmu).

## 2. Our Proposed Approaches

As a special language, sign language has its own specific linguistic rules as well [10], so the SLT model follows the NMT framework, as shown in Figure 1. Now suppose that **y**=(*y*_1_, *y*_2_,…, *y*_*T*_*y*__) is an output sentence that corresponds to the sign video frame sequence **x**=(*x*_1_, *x*_2_,…, *x*_*T*_*x*__) in the training set. At the very beginning, we use the unsupervised *FSDC* algorithm module to optimize the frame-level input sentences. Then, a spatial CNN is used to convolute frames to gain tokenization layer which is then input into the NMT module for encoding and decoding. In this section, we will introduce the proposed approaches in detail.

### 2.1. Unsupervised *FSDC* Module

**Figure 2:**
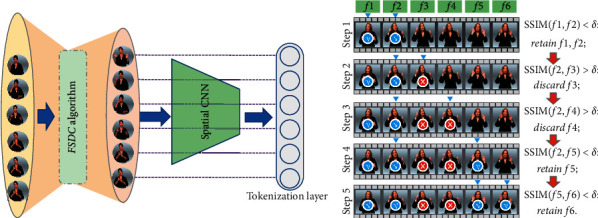
(a) The spatial CNN part with the proposed *FSDC* algorithm module. (b) Scaled *FSDC* module with a running example.

As shown in Figure 2(a), the spatial CNN is mainly used to reduce the fine-grained input of video frames. In SLT, the video frame is the most basic input unit. The compression of video frames directly affects the processing efficiency of CNN and the quality of the tokenization layer. Therefore, optimizing the number of frames also means optimizing the tokenization layer.

For any video dataset, we must follow a fixed *frames per second* (FPS) to frame all the videos, which leads to massive similar redundant frames in the temporal neighborhood. As an illustration, a signer signs the same sign language at fast and slow speeds, respectively. Although the two express the same meaning, they produce videos of different lengths. Obviously, a video signed at a slower speed will get more redundant similar frames in temporal neighborhood.

To reduce this effect, the *FSDC* algorithm is proposed. We delete the less-important frames by comparing the similarity index and to keep the sequence of the frames fixed at the same time. In theory, it helps us to reduce the amount of training data as well as errors caused on account of sign speed and *FPS*.

**Figure 3:**
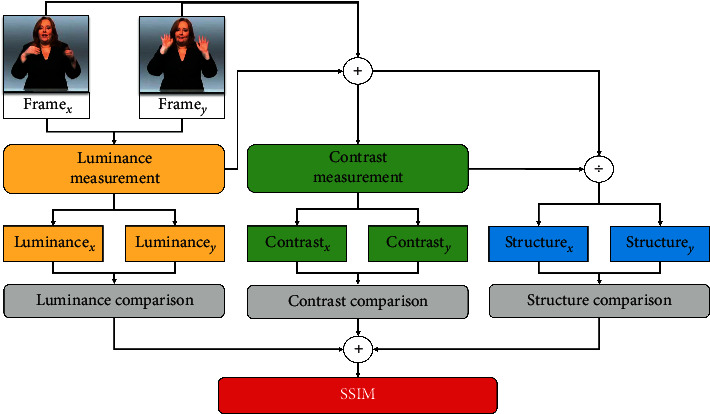
The process of comparing SSIM values between two images.

We use the SSIM algorithm [11] to calculate the similarity between two images, which is close to the intuitive feeling of the human eye. When calculating the structural similarity of frame *f*_*i*_ and frame *f*_*j*_, the corresponding calculation flow chart is shown in Figure 3. The formula of the SSIM algorithm is as follows:

(1) $$\begin{array}{c} \text{SSIM}\left(f_{i},f_{j}\right)={\left[L\left(f_{i},f_{j}\right)\right]}^{x}\cdot {\left[C\left(f_{i},f_{j}\right)\right]}^{y}\cdot {\left[S\left(f_{i},f_{j}\right)\right]}^{z}, \end{array}$$

where *L*(^∗^) denotes the luminance comparison, *C*(*∗*) denotes the contrast comparison, and *S*(*∗*) denotes the structure comparison. Note that *x* > 0, *y* > 0, and *z* > 0, we initialize *x*=*y*=*z*=1. SSIM(*∗*) is a decimal between 0 and 1. Extremely, SSIM=1 means two images are completely identical, while SSIM=0 means completely different.

The *FSDC* calculates the SSIM indexes for both each frame and all frames in the neighborhood. If the SSIM index is greater than a certain threshold *δ*(0 < *δ* < 1), only one of them will be retained, while the rest will be discarded as redundant frames. A running example of Algorithm 1 is shown in Figure 2(b).

Formally, we explore frame-level input tokenization as shown in Figure 2(a) and map the feature vectors to the tokenization layer as

(2) $$\begin{array}{c} \Gamma =\text{SpatialCNN}\left(FSDC\left(x\right)\right). \end{array}$$

### 2.2. TC-DHBG-Net for Encoding Stage

**Figure 4:**
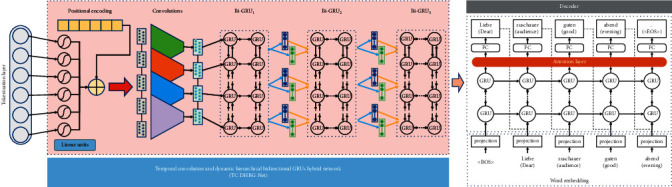
The improved encoder in the NMT module with a TC-DHBG-Net.

Figure 4 shows the improved NMT module we proposed. Specifically, we improve the encoder in two folds. The first is *T-Conv* unit for the tokenization layer; and the second is *DH-BiGRUs* for mining semantic information.

The T-Conv unit is inspirited by the work of Bérard et al. [12] on the end-end speech task. It takes as input a sequence of features for tokenization layer. These features are given as input to two nonlinear (tanh) layers, which output new features of size *n*. In order to enhance the optical flow feature capture, we concatenate the positional encoding [13] to obtain the feature vectors with position information. Like [14], this new set of features is then passed to a stack of two convolutional layers. Each layer applies 16 convolution filters of shape (3, 3, depth) with a stride of (2, 2) w.r.t. time and feature dimensions; depth is 1 for the first layer and 16 for the second layer. We get features of shape (*T*_*x*_/2, *n/*2, 16) after the 1st layer and (*T*_*x*_/4, *n*/4, 16) after the 2nd layer. This latter tensor is flattened with shape (*T*_*x*_ = *T*_*x*_/4, 4*n*) before being passed to a stack of three-level *DH-BiGRUs*. This set of features has 1/4th the time length of the initial features, which speeds up the raining because the complexity of the model is quadratic with respect to the source length.

The DH-BiGRU unit computes a sequence of annotations *h*=*h*_*i*_,…, *h*_*T*_*x*__, where each annotation *h*_*i*_ is a concatenation of the corresponding forward and backward states. The hidden state of the last GRU layer in each hierarchy is inserted into the next hierarchy. Formally, first we insert the tokenized vectors into a recurrent neural structure to obtain the semantic information of the context sequence. For recurrent unit type, we choose GRU [15] instead of LSTM [16] because the former has fewer gate structures. The hierarchical structure [2, 12, 17] and bidirectional structure can extract deeper relevant information. Suppose that the hierarchy of HGRU is *n*, then

(3) $$\begin{array}{c} \xi_{\text{encoder}}=\varphi_{en\_rnn_{n},en\_rnn_{n-1},\ldots ,en\_rnn_{1}}\left(\Gamma \right)=\left(h_{1},h_{2},\ldots ,h_{n^{\prime }}\right), \end{array}$$

where (*h*_1_, *h*_2_,…, *h*_*n*′_) are the hidden states of the last GRU layer, and *n*′ is a variable, and *φ*_*en*_*rnn*_(^∗^) indicates the processing of RNN in the encoder.

### 2.3. Decoder and Attention Mechanism

#### 2.3.1. Decoder

For the word embedding, we use a fully connected layer that learns a linear projection from one-hot vectors of spoken language words to a denser space as follows:

(4) $$\begin{array}{c} \omega_{i}=\text{WordEmbedding}\left(y_{i}\right), \end{array}$$

where *ω*_*i*_ is the embedded version of the spoken word *y*_*i*_.

In the decoding stage, we aim at maximizing the probability *p*(**y**|**x**). The decoder computes a probability of the translation **y** by decomposing the joint probability into the ordered conditional probabilities as follows:

(5) $$\begin{array}{c} p\left(\left.y\right|x\right)=\overset{T_{y}}{\underset{i=1}{\prod }}p\left(\left.y_{i}\right|y_{1},y_{2},\ldots ,y_{i-1},\left(h_{1},h_{2},\ldots ,h_{n^{\prime }}\right)\right). \end{array}$$

#### 2.3.2. Attention Mechanism

Like other SLT models, we may also suffer from long-term dependencies, vanishing gradients, and performance deterioration with many input frames. To solve the issues, we utilize attention mechanisms which have been proved useful in various tasks including but not limited to machine translation. The most common attention mechanisms are the mechanisms of Bahdanau et al. [18] and Luong et al. [19]. Based on hyperparameter experiments, we take Bahdanau as our attention mechanism. Given the input **x**, we define each conditional probability at time *i* depending on a dynamically computed context vector *c*_*i*_ as follows:

(6) $$\begin{array}{c} p\left(\left.y_{i}\right|y_{1},y_{2},\ldots ,y_{i-1},x\right)=\text{soft}\max\left(g\left(s_{i}\right)\right), \end{array}$$

where *s*_*i*_ is the hidden state of the decoder at time *i* and *g* is a linear transformation that outputs a vocabulary-sized vector. Note that the hidden state *s*_*i*_ is computed as

(7) $$\begin{array}{c} s_{i}=\varphi_{de_{rnn}}\left(\omega_{i-1},s_{i-1},\omega_{i}\right), \end{array}$$

where *φ*_*de*_*rnn*__(^∗^) indicates the processing of RNN in the decoder and *ω*_*i*−1_ is the word embedding of the previously predicted word *y*_*i*−1_, *s*_*i*−1_ is the last hidden state of the decoder, and *c*_*i*_ is computed as a weighted sum of the hidden states from encoder as

(8) $$\begin{array}{c} c_{i}=\sum_{j=1}^{T_{y}}\alpha_{ij}h_{j}, \end{array}$$

where *α*_*ij*_ is the weight of each annotation *h*_*j*_.

## 3. Experiments

In this section, we conducted a series of experiments on the *RWTH-PHOENIX-Weather 2014T* dataset by employing our improved SLT model with tokenization-related units compared to the baseline.

### 3.1. Baseline

As described above, the baseline is an attention-based structure combined by 2DCNN and Seq2Seq sequentially. The spatial 2DCNN is an AlexNet [20], and its parameters are pretrained on Imagenet [21]. The encoder and decoder of Seq2Seq are nonhierarchical GRUs. In order to compare with the baseline fairly, all experiments run in the same dataset and GPU environment. Except for the differences mentioned in the paper, other configurations for all models are consistent by default.

### 3.2. Dataset

The *RWTH-PHOENIX-Weather 2014T* is the most popular continuous SLT dataset. It is collected by extending the German sign language recognition (SLR) dataset, *RWTH-PHOENIX-Weather 2014 Corpus* [22]. Compared with other SLT datasets, this dataset has larger data and higher quality. It contains 4,839 vocabulary, 8,257 video clips, 947,756 frames, and 113,717 words in total, as shown in Table 1. Each video corresponds to a translation sentence. Although the dataset includes sign language gloss corpus, our model is trained without gloss-level alignment, where the glosses give the meaning and the order of signs [1, 23, 24]. Nevertheless, the use of glosses is limited to a prerequisite that word label in sentences is consistent with the order of corresponding visual content. In the other words, if the word is out of order, it is unsuitable to tackle sequential frame-level classification under word labels in disorder. In fact, most datasets do not include gloss annotations. Although we do not consider it for this work, we conducted NMT experiments using gloss to gain optimal settings as [1].

### 3.3. Settings

Based on baseline conclusions and our experience, we preset some important hyperparameters. We use GRU as the recursive module for both encoder and decoder, where each recurrent layer contains 1,000 hidden units. During the training, the optimizer used is Adam [25], and the learning rate is 0.00001 with a decay factor of 0.98 and a batch size of 1. During the decoding, we use *beam search* with a width size of 3 to generate sentences.

### 3.4. Evaluation

We use BLEU [26] and ROUGE [27] as the evaluation metrics, which are most used in machine translation tasks. Note that the BLEU score is represented by BLEU-1, 2, 3, 4 and the ROUGE score refers to ROUGE-L F1-SCORE. In training, the BLEU-4 score on the development set is used to select the best model.

### 3.5. Comparison to Existing Approaches

Table 2 shows the performance comparison between our proposed systems and the existing baseline systems.

The existing baseline systems use different attention mechanisms, of which the Bahdanau mechanism performs best. It is worth mentioning that although the transformer has good performance in many NMT tasks, it does not achieve good results in the SLT dataset due to its small data size.

Our proposed systems contain innovations in multiple places, so we added different improved modules on the baseline for comparison. We can see that after using the unsupervised *FSDC* algorithm (#2h), the model achieves better performance. As for the improvement of the encoder in NMT module, either *T-Conv* or *DH-BiGRUs* units have a promoting effect as shown in Table 2 (#2e and #2f). The complete improved encoder module which uses both *T-Conv* and *DH-BiGRUs* units (i.e., TC-DHBG-Net) improves more significantly as shown in Table 2 (#2g). From the performance, we can see that the improved encoder in the NMT module is the most important and the *FSDC* algorithm can slightly improve the basis as shown in Table 2 (#2i). Overall, the proposed tokenization-related units without extra information improve significantly for the SLT.

### 3.6. Validation on TC-DHBG-Net

In order to validate the role of the *T-Conv}* unit of the *TC-DHBG-Net*, we only add *T-Conv* units to the encoder of the baseline, while the recursive neural unit remains unchanged. In Table 2, #2e exceeds the baseline moderately, which proves the positive role of the *T-Conv* unit.

The *DH-BiGRUs* unit is another important component of the *TC-DHBG-Net*. We replace the original GRUs of the baseline with our *DH-BiGRUs* unit in 3 levels by default. As shown in Table 2 (#2f), the multilevel structure is introduced and the performance is moderately improved, proving the effect of the hierarchical structure.

Although *T-Conv* unit and *DH-BiGRUs* unit have been proved by the above experiments, it does not mean that the combination of the two will be better. Therefore, it is necessary to introduce Table 2 (#2g). Compared with baseline, #2g improves significantly, which is better than any single module (#2e or #2f).

### 3.7. Ablation on the Levels of DH-BiGRUs

The *DH-BiGRU* has an important hyperparameter, the number of RNN levels. To test the scores for different levels of *DH-BiGRU* in the recurrent neural unit, we set the number *N*_level_ to 1, 2, 3, and 4, respectively. We conducted experiments based on the previous experiment as shown in Table 2 (#2g).  Table 3 illustrates that the hierarchical structure has a significant impact on the scores. When *N*_level_ is set to less than 3, the scores increase as the number of levels increases, and when *N*_level_=3, the score increases to peak; but when *N*_level_ > 3, the score starts to drop. As a conclusion, a larger number of layers do not mean a higher score. Therefore, we set *N*_level_=3 to the optimal hyperparameter.

### 3.8. Validation on *FSDC* Algorithm

**Figure 5:**
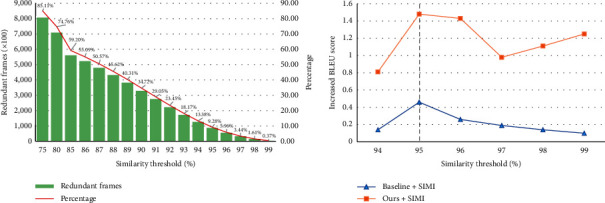
(a) Numbers and percentage of redundant frames with respect to different similarity thresholds. (b) The increased absolute values of BLEU compared to the baseline after using the *FSDC* algorithm. When the threshold is around 95%, both models reach the peak.

At the very beginning, we analyze the structural similarity of all frames in the dataset.  Figure 5(a) shows that the number or proportion of the separable redundant frames varies with different thresholds. Even if the threshold is set to 75%, we can see that the number of frames for temporal neighborhood exceeds 85%. Once the threshold is lower, the proportion of frames will be greater. This indicates that the relationship between the frames is tight. A reasonable initial threshold is crucial to the model, but the threshold is an empirical and experimental hyperparameter. If the threshold is set too low, much more useful frame information may loss; on the contrary, the optimization will not work at all. Analyzing Figure 5(a), we think that the similarity threshold is set to at least 94%.

To validate the *FSDC* algorithm, we set the thresholds from 94% to 99%, to control the percentage of redundant frames. We conducted the experiment on the baseline (Table 4 (#4a)) and the structure we proposed (Table 4 (#4b)), respectively.  Figure 5(b) shows that within a reasonable range, the *FSDC* algorithm can be positive relative to the improvement of the baseline, especially when the threshold is set to 95%. But the relative value of negative numbers in Table 4 (#4b) also shows that not all thresholds can improve performance.

Moreover, it is worth mentioning that the size of the training data is reduced by 9.28% when the threshold is set to 95%. The optimized dataset not only saves storage space, but also saves processing time (about 10% reduction).

### 3.9. About Length

**Figure 6:**
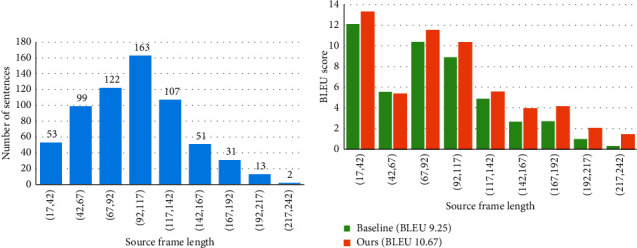
(a) Numbers and percentage of redundant frames with respect to different similarity thresholds. (b) The increased absolute values of BLEU compared to the baseline after using the *FSDC* algorithm. When the threshold is around 95%, both models reach the peak.

Figure 6(a) shows the distribution of the number of sentences with respect to the different lengths of source sentences (frames) on the test set. Since the frame number of most sentences is less than 100, we think that more than 100 frames are considered as long sentences.  Figure 6(b) shows the BLEU scores of generated translations on the test set with respect to the lengths of the source sentences. In particular, we split the translations into different bins according to the length of source sentences (frames), and then test the BLEU scores for translations in each bin separately with the results reported in Figure 6(b). Our approach can achieve big improvements over the baseline system in almost all bins, especially in the long sentences which have more than 117 frames. The performance comparison intuitively shows that our model can better adapt to the translation of long sentences, which benefits the *FSDC* algorithm and the improved encoder.

### 3.10. Qualitative Comparison

As shown in Table 5, to help readers understand our translations better, we qualitatively analyze the results of the sentence-level experiments. The sentences shown in the examples are both long sentences. The frame numbers of examples (a) and (b) are 192 and 196 frames, respectively. After using our *FSDC* optimization algorithm, the frame numbers are reduced to 182 and 169 frames, respectively. Since long sentences have serious long-distance dependency problems, both examples show that the current SLT models have poor translation ability to deal with long sentences. Comparing the baseline and our model, our model is relatively more accurate, and the meanings of the sentences are closer to the ground true. Note that the translation results closer to the target in Table 5 are marked in bold.

## 4. Related Work

According to a recent review [28], sign language is an ongoing research that began decades ago. The SLR system can be classified into three based on the type: (1) fingerspelling recognition; (2) isolated word recognition; (3) continuous sign sentence recognition. As for SLT, it is a more advanced task to further understand the semantic information of sign language.

In earlier work, the SLR system employed traditional recognition methods. For instance, Gao et al. [29] used HMM to recognize SLR words; The authors of [30, 31] used SVM to classify continuous sign language alphabets and isolated words; Baccouche et al. [32] performed a trajectory matching to classify the isolated words. Compared to the above, deep learning-based models have been employed recently. CNNs [33, 34], LSTMs [2, 35–37], or hybrid models [3, 38] have been used for continuous sentence recognition.

When it comes to SLT, few research results are published up to now. However, the development of SLR has laid a foundation for SLT. Camgoz et al. [1] released the first available continuous SLT dataset and proposed a neural SLT model. They combined CNN with the classic machine translation model-Seq2Seq. Their work maintains state of the art on the RWTH-PHOENIX-Weather 2014T dataset. Later, Ko et al. [4] proposed a neural SLT model based on human pose estimation, converting a video frame to keypoints, which simplifies the complexity of recognition, but ignored much important semantic information, e.g., expressions. We believe that it is under consideration. Guo et al. [2] proposed a hierarchical LSTM model that performed both SLR and SLT experiments on a Chinese dataset. They used 3DCNN for features extraction and compared it with the video captioning model S2VT [39]. The critical problem about their dataset is that it only includes 100 sentences, which is inappropriate for translation tasks. Overall, SLT achievement is still underperforming, limited by a lack of large-scale datasets and better translation models.

## 5. Conclusion

In this work, we propose a novel weakly supervised SLT model with improved tokenization-related modules to adapt to longer sentences. We first propose an *FSDC* algorithm for temporal neighborhood to optimize the limited training data by removing the redundant frames and compress the sentence length to get a better interpretability. Then we introduce a *T-Conv* and *DH-BiGRU*-mixed NMT, which can consider the temporal information with reasonable resource consumption as well as succeed in extracting deeper information. To evaluate our approaches, we conducted experiments on the public dataset-*RWTH-PHOENIX-Weather 2014T*. Compared with the existing state-of-the-art baseline, our model can reduce the size of training data by **9.3%** and outperform the baseline up to about **1.5+** BLEU-4 score on the sign-to-text translation task. Moreover, we conducted a series of comparison and ablation experiments and analyzed the translation performance qualitatively.

Despite the improved performance, SLT still has a lot of room to be studied. In future work, we will explore better interpretative methods to translate longer sentences.

## Figures and Tables

**Algorithm 1:**
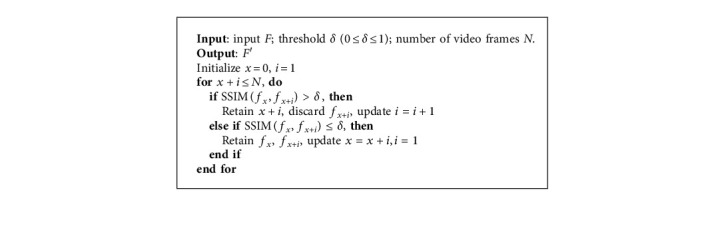
*FSDC* algorithm for temporal neighborhood.

**Table 1:** Key statistics of the German datasets.

	Train	Dev	Test
Vocab.	2,887	951	1,001
Clips	7,096	519	642
Frames	827,354	55,775	64,627
Tot. words	99,081	6,820	7,816

**Table 2:** Experiments on the existing baseline systems vs. variants of our novel model.

#	Model	Development set					Test set				
		ROUGE	BLEU-1	BLEU-2	BLEU-3	BLEU-4	ROUGE	BLEU-1	BLEU-2	BLEU-3	BLEU-4
*Existing baseline systems*											
2a	None	29.54	28.33	15.71	10.32	8.57	28.60	26.65	15.02	10.27	8.24
2b	Transformer	30.28	29.82	16.98	11.89	8.93	29.89	29.45	16.72	11.78	8.82
2c	Luong	31.67	**32.18**	18.56	12.38	9.46	30.71	30.01	17.43	12.11	9.02
2d	Bahdanau	**31.93**	31.66	**18.70**	**12.79**	**9.53**	**31.56**	**31.32**	**18.36**	**12.36**	**9.25**

*Our proposed systems*											
2e	+T-Conv	32.08	30.08	18.15	12.88	9.97	31.34	30.94	18.26	12.71	9.76
2f	+DH-BiGRUs	31.55	30.21	18.29	13.05	9.84	31.20	31.46	17.64	12.40	9.65
2g	+TC-DHBG-Net (+T-Conv + DH-BiGRUs)	31.69	31.23	18.62	13.15	10.16	32.25	**32.19**	19.38	13.71	10.66
2h	+*FSDC*	32.13	**31.72**	18.84	12.98	9.79	31.52	31.72	19.04	13.01	9.71
**2i**	**+** *FSDC* **+** TC-DHBG-Net	**32.76**	31.43	**19.12**	**13.40**	**10.35**	**32.99**	31.86	**19.51**	**13.81**	**10.73**

Bold indicates the best performance.

**Table 3:** BLEU scores on *DH-BiGRU* unit in different levels.

#	Levels	Development set					Test set				
		ROUGE	BLEU-1	BLEU-2	BLEU-3	BLEU-4	ROUGE	BLEU-1	BLEU-2	BLEU-3	BLEU-4
3a	1	31.34	30.94	18.26	12.71	9.76	32.18	31.60	18.52	12.43	9.52
3b	2	31.69	31.23	18.62	13.15	10.16	32.08	30.08	18.15	12.88	9.97
3c	3	**33.02**	**32.37**	**19.49**	**13.44**	**10.21**	**32.25**	**32.19**	**19.38**	**13.71**	**10.66**
3d	4	31.52	31.40	18.71	13.00	9.87	31.58	31.85	18.95	13.17	10.03

**Table 4:** BLEU scores vary in different thresholds.

#	Thresholds	94	95	96	97	98	99	100
**4a**	Baseline	—	—	—	—	—	—	9.25
	+*FSDC*	9.39	**9.71**	9.51	9.44	9.39	9.35	—
	△	+0.14	**+0.46**	+0.26	+0.19	+0.14	+0.10	—

**4b**	+Ours	—	—	—	—	—	—	10.66
	+Ours + *FSDC*	10.06	**10.73**	10.68	10.23	10.36	10.50	—
	△	+0.81(−0.60)	**+1.48 (+0.07)**	+1.43 (+0.02)	+0.98 (−0.43)	+1.11 (−0.30)	+1.25 (−0.16)	—

△ represents the increased absolute values of BLEU from the baseline, and the scores in parentheses represent the relative change value from +Ours. The *FSDC* algorithm does not work when the threshold is 100%.

**Table 5:** Comparison of translations between our model and baseline.

**Example (a)**	
Source	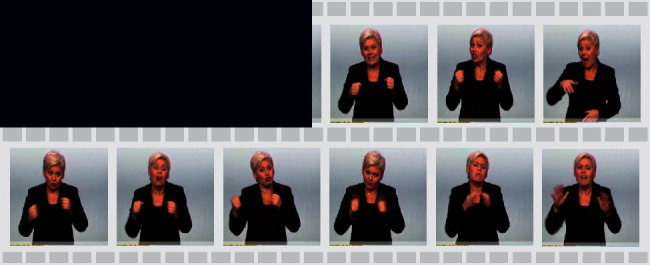
Target	der wind weht mäßig bis frisch mit starken bis stürmischen böen im bergland teilweise schwere sturmböen im südosten mitunter nur schwacher wind. (The wind blows moderately to fresh with strong to stormy gusts in the mountains, sometimes severe gusts in the southeast, sometimes only weak winds.)
BASE	der wind weht mäßig im norden frisch mit frisch mit stürmischen böen an der nordsee schwere sturmböen. (**The wind blows moderately** in the north fresh with fresh with stormy gusts at the north sea heavy gusts of wind.)
OURS	der wind weht mäßig bis frisch bei schauern und gewittern kann es stürmische böen auf den bergen sturmböen. (**The wind blows moderately to fresh** during showers and thunderstorms, it can be **stormy gusts on the mountains**.)
Frames	From 192 to 182

**Example (b)**	
Source	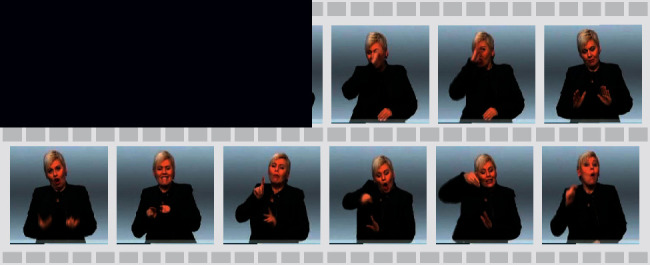
Target	und morgen wird es dann in der südosthälfte nochmal ähnlich werden wie heute allerdings im nordwesten bereits dichtere wolken. (and tomorrow it will be similar again in the southeast half of the day as in the northwest, however, with thicker clouds.)
BASE	morgen im süden und süden bleibt es allerdings schon wolkenlücken und gewitter das wird es schon schon werden werden aus den westen. (**Tomorrow** in the south and south there will be cloud gaps and thunderstorms it will be from the west.)
OURS	und morgen wird es dann in der südosthälfte nochmal ähnlich am alpenrand wieder mal südwestwind und gewitter. (**and tomorrow it will be similar in the south-east half again** on the edge of the alps again south-west wind and thunderstorm.)
Frames	From 196 to 169

**BASE**: baseline model; **Ours**: the optimal model mentioned above; and the texts in parentheses represent the English translation corresponding to German.

## Data Availability

The data we use can be accessed at https://www-i6.informatik.rwth-aachen.de/∼koller/RWTH-PHOENIX-2014-T/.
